# Optimised blended treatment for alcohol use disorder (OptiBlend) in Dutch outpatient addiction care: study protocol for a three-arm randomised controlled trial

**DOI:** 10.1136/bmjopen-2026-127286

**Published:** 2026-11-06

**Authors:** Lisa Elze Imhof, Puck Theresa Gerarda Maria Veldman, Eline Josephina Maria Bol, Nick Marin Lommerse, Anique Caroline Jansen, Johanna Carlijn Quinta de Vries, Milou van Paridon, Anna Emma Goudriaan, Matthijs Blankers

**Affiliations:** 1 Jellinek, Arkin Mental Health Care, Amsterdam, The Netherlands; 2 Department of Research, Arkin Mental Health Care, Amsterdam, The Netherlands; 3 Department of Psychiatry, Amsterdam University Medical Centers, Amsterdam, The Netherlands; 4 University of Amsterdam, Amsterdam, The Netherlands; 5 Trimbos Institute, Netherlands Institute of Mental Health and Addiction, Utrecht, The Netherlands; 6 Tranzo Scientific Center for Care and Wellbeing, Tilburg School of Social and Behavioral Sciences, Tilburg University, Tilburg, The Netherlands

**Keywords:** PSYCHIATRY, Randomized Controlled Trial, Adolescent

## Abstract

**Introduction:**

Alcohol use disorder (AUD) is highly prevalent and associated with substantial personal and societal costs, and a large treatment gap. Although traditional forms of treatment are effective, relapse rates remain high and treatment adherence is suboptimal. Digital interventions can improve access to care, but limited therapist contact may reduce the strength of the therapeutic alliance. Blended treatment, combining therapy sessions with structured guided digital modules within a single treatment protocol, aims to integrate the strengths of both approaches. However, evidence regarding its clinical and cost-effectiveness and the optimal number of therapy sessions compared with traditional treatment in AUD remains limited. The OptiBlend study described in this protocol aims to evaluate the effectiveness and cost-effectiveness of optimised blended treatment for AUD.

**Methods and analysis:**

The study is a three-arm, pragmatic, single-blinded randomised controlled trial conducted at four outpatient facilities of a large addiction care institution in the Netherlands. Patients with AUD are randomised to one of two optimised forms of blended cognitive behavioural therapy (CBT) treatment with individual therapy (in person or video call) sessions: Blended-13 (13 sessions) or Blended-8 (8 sessions), or to treatment as usual (TAU; 13 sessions). Both blended interventions include structured guided digital modules and additional lifestyle modules targeting nutrition and sleep. In TAU, CBT treatment modules are delivered using a printed workbook. The primary outcome is weekly alcohol consumption at 12-month follow-up, assessed using Timeline Follow-back methodology. Data are collected through self-report questionnaires, ecological momentary assessment and usage data from the digital treatment platform. Treatment effects will be estimated using linear mixed-effects models.

**Ethics and dissemination:**

The study protocol has been approved by the Medical Research Ethics Committee Amsterdam UMC (METC Amsterdam UMC: NL84117.018.23). All participants provide written informed consent before study participation. Findings will be disseminated through peer-reviewed publications, conference presentations and lay summaries for participants and the general public.

**Registration:**

The trial was pre-registered on 17 October 2024 on onderzoekmetmensen.nl.

**Trial registration number:**

onderzoekmetmensen.nl (NL-OMON57089).

**STRENGTHS AND LIMITATIONS OF THIS STUDY**

- This is a three-arm, pragmatic, single-blinded randomised controlled trial conducted in a real-world addiction care setting, enhancing the ecological validity of the findings.
- The study compares two forms of blended treatment with different numbers of individual therapy sessions, allowing evaluation of whether treatment intensity can be optimised while maintaining effectiveness.
- Patients and therapists were actively involved in the development of the optimised blended treatment modules, supporting a patient-centred design and enhancing clinical relevance.
- Participants and therapists cannot be blinded to treatment allocation, which may introduce bias, although outcome assessors are blinded.
- The use of self-reported outcome measures, potential variation in treatment adherence and potential protocol deviations may introduce measurement bias and affect internal validity.

## Background

 Alcohol use disorder (AUD)^1^ is a highly prevalent mental health condition with substantial consequences for individuals and society. Globally, approximately 7% of adults meet the criteria for AUD each year, while in the Netherlands, an estimated 5.4% of the population is affected.^2 3^ AUD accounts for over 19.3 million disability-adjusted life years lost annually, reflecting a considerable societal and economic impact.^2^ In addition, AUD is associated with disruptions in lifestyle domains, such as nutrition and sleep, which contribute to psychological distress and relapse in AUD.^4–8^

There is a broad consensus regarding effective treatment for AUD. Cognitive behavioural therapy (CBT) and motivational interviewing (MI) are recommended in both national and international clinical guidelines^9 10^ and have consistently been shown to be effective in reducing alcohol use.^11–14^ Traditionally, these interventions are delivered through sessions with a therapist.

Despite their efficacy, traditional forms of treatment have important limitations. A substantial proportion of individuals do not seek or receive treatment^15^ with barriers including stigma, logistical constraints (eg, treatment offered only during working hours), high treatment costs and perceived mismatch between available treatments and personal needs.^16–18^ Access to care is further restricted by long waiting lists and limited treatment capacity, partly due to shortages of trained healthcare professionals in mental healthcare.^19–21^ Moreover, for those who start treatment, relapse rates are high, and treatment adherence is often suboptimal.^22 23^ Together, these limitations highlight the need for more accessible and efficient treatment approaches for AUD.

Digital psychological treatments offer the possibility to address these limitations by reducing practical barriers and improving access to care.^24–26^ Broadly, three types of digital interventions are distinguished in the literature: unguided, guided and blended interventions. Unguided digital interventions typically consist of self-help programmes without therapist involvement, while guided digital interventions include some degree of support from a therapist or coach. Blended interventions integrate (guided) digital components with therapy sessions.^27^

Evidence shows that unguided digital self-help interventions can reduce alcohol consumption in non-treatment-seeking or subclinical populations^24 28 29^ but low adherence and dropout are common due to the lack of therapist support.^30^ Guided digital interventions demonstrate better treatment outcomes and adherence compared with unguided interventions.^30 31^ Although guided digital interventions can foster a therapeutic alliance, their depth and reciprocity are often limited compared with therapy sessions.^32^

Blended treatment combines the strengths of both digital and traditional therapy approaches within a single treatment protocol.^27 33 34^ In blended treatment, therapy sessions, conducted in person or via video call, are complemented by guided digital modules. Inclusion of therapist contact enhances motivation to complete treatment,^35 36^ while digital components allow patients to work independently and apply skills in everyday contexts. Through the digital platform, therapists can monitor progress, give feedback and guide patients remotely, enabling parts of the treatment to occur without direct therapist contact.^35 37^ A recent review and meta-analysis found that adding digital support to treatment is effective, whereas partial replacement of treatment does not appear to worsen outcomes, although evidence remains limited.^38^ In this way, the blended format may offer the potential to reduce the number of required therapy sessions, thereby reducing reliance on trained healthcare professionals and saving treatment costs while maintaining clinical effectiveness.^19–21 39^

Relapse is a persistent challenge in AUD, particularly within the first year after treatment,^40^ yet structured continuing care is often lacking. Evidence suggests that continuing care, by reinforcing coping strategies and skills to manage high-risk situations, can support relapse prevention in line with Marlatt’s model.^41^ This is especially the case when continuing care involves active participation, as in digital interventions.^42–44^ In blended treatment, continuing care can be integrated through post-treatment modules that target relapse risk and support the maintenance of abstinence or controlled alcohol use.

To further reduce the risk of relapse and lower psychological distress, blended treatment can also address modifiable lifestyle factors like nutrition and sleep that are associated with negative outcomes in AUD.^45 46^ Nutritional deficiencies can worsen mood and anxiety symptoms, while sleep disturbances are linked to both the onset and recurrence of AUD.^4 7 45 47^ Although effective interventions exist, such as nutrition education and CBT for insomnia,^47–51^ they are not systematically integrated into AUD care. Digital modules targeting these relapse-related lifestyle factors can therefore be incorporated into blended treatment to enhance outcomes.

Blended treatment is already implemented in clinical practice, including at large addiction care institutions such as Jellinek in the Netherlands, using structured protocols based on CBT and MI. Implementation studies show positive attitudes among AUD patients and therapists,^34 52 53^ and evidence in other psychiatric disorders suggests that blended treatment is more effective than traditional treatment as usual (TAU) and may reduce dropout and save clinician time.^27 34 35 54–57^ However, evidence for the effectiveness of blended treatment in AUD remains limited. To our knowledge, only one other randomised controlled trial (RCT) to date has been initiated to evaluate the (cost-)effectiveness of blended treatment in AUD.^58^ Moreover, the potential impact of reducing the number of therapy sessions has not yet been examined.

To address these gaps, we developed the ‘Optimising Blended Interventions and Aftercare for Alcohol Addiction’ (OptiBlend) study. In collaboration with patients and therapists, we optimised the existing blended modules offered at Jellinek and developed new modules focused on nutrition and sleep. This protocol paper presents our three-arm, single-blind RCT at Jellinek to evaluate the effectiveness and cost-effectiveness of this optimised blended treatment. In the OptiBlend trial, we compare individual protocolised CBT TAU (13 therapy sessions) with two forms of optimised blended treatment: one with the same number of individual therapy sessions as TAU (Blended-13) and one with a reduced number of 8 therapy sessions (Blended-8). Ecological momentary assessment (EMA) is used to evaluate the additional effects of nutrition and sleep modules on dietary habits, sleep quality and alcohol use.

We hypothesise that:

1. Blended treatment (Blended-13+Blended-8) will be more effective in reducing weekly alcohol consumption at the 12-month follow-up (primary outcome) compared with TAU (Cohen’s *d*≥0.35).
2. Blended-8 will be non-inferior to Blended-13 in reducing weekly alcohol consumption at the 12-month follow-up, using a non-inferiority margin of 10%.
3. Blended-8 will be more cost-effective than both Blended-13 and TAU, from both a healthcare sector and broader societal perspective (20% lower AUD treatment costs in Blended-8).
4. Blended-13 and Blended-8 will show greater improvements in healthy nutrition and sleep than TAU (EMA study—Cohen’s *d*≥0.60).

## Methods

The trial was preregistered on 17 October 2024 on onderzoekmetmensen.nl (NL-OMON57089). The reporting of this study protocol is in accordance with the Standard Protocol Items: Recommendations for Interventional Trials (SPIRIT) guidelines.^59^

### Study design

The OptiBlend study is a pragmatic, single-blind RCT with three parallel treatment arms of equal group sizes. Participants are randomly allocated in a 1:1:1 ratio to one of three treatment arms (Blended-13, Blended-8 or TAU). Randomisation is performed using Castor Electronic Data Capture (EDC) with variable block sizes of 6, 9 and 12. Randomisation is stratified by trial site to ensure balance across the four outpatient locations from which Jellinek participants are recruited: Amsterdam, Utrecht, Hilversum and Amersfoort (the Netherlands). Jellinek is part of Arkin, an integrated mental healthcare organisation in the Netherlands. Before randomisation and allocation to one of the three treatment arms of the RCT, participants are invited to participate in the add-on EMA study. Therefore, equal distribution of EMA participants across the treatment arms is expected.

The RCT follows a single-blind design, in which research assistants conducting follow-up assessments are blinded to the condition to which patients have been allocated, to reduce bias in data collection. Given the nature of the interventions (eg, the use of digital materials in Blended-13 and Blended-8, and the absence of digital materials in TAU), blinding of participants and therapists is not deemed feasible. Participants and therapists are explicitly instructed not to disclose the allocation outcome to the blinded research assistants. Blinding is maintained through predefined user roles and permission settings in Castor EDC. The research assistants will not be unblinded under any circumstances. Junior researchers involved in trial coordination are not blinded as they manage day-to-day study procedures and communicate randomisation outcomes to participants and therapists through a separate email inbox. Therefore, they are not involved in conducting follow-up assessments.

### Eligibility criteria

Patients are deemed eligible to participate in the OptiBlend trial if they meet the following criteria: (1) primary diagnosis of AUD according to the DSM-5 criteria, as assessed by their therapist at Jellinek; (2) indication for receiving blended outpatient treatment after the intake at Jellinek; (3) voluntarily enrolled in outpatient AUD treatment at one of the participating sites; (4) access to a smartphone, tablet or computer at home; (5) at least 18 years of age and (6) at least moderate proficiency in Dutch. Exclusion criteria are the following: (1) indication for inpatient AUD treatment, (2) indication for involuntary or forensic care, (3) presence of an acute psychosis and (4) diagnosis of dementia.

Treatment sites are eligible to participate in the study if they offer Jellinek’s blended treatment programme as part of routine care. The therapists who offer blended treatment are psychologists supervised by a licensed healthcare psychologist. Before a therapist can participate in the study and administer the interventions, they need to complete training on applying the Blended-8, Blended-13 and TAU protocols and attend a briefing on the study procedures.

### Intervention and comparator

Patients are randomised to one of three treatment conditions. The experimental conditions are two forms of optimised blended treatment: one with standard therapy session intensity (Blended-13) and the other with reduced therapy session intensity (Blended-8), and the control condition is TAU. In all conditions, therapy sessions are delivered by a therapist, either in person or via video call. TAU was selected as a comparator because it reflects the current standard of care for patients with AUD within Jellinek. Including TAU as an active control group enables a pragmatic evaluation of the added value and clinical effectiveness of the blended treatment formats (Blended-13 and Blended-8) compared with routine care.

All treatment conditions in the OptiBlend trial are based on standard interventions provided at Jellinek. Consequently, participation in the OptiBlend trial is not expected to pose any additional clinical risk to participants. Therefore, adverse events will not systematically be recorded. Serious adverse events will be reported by the investigator to the sponsor (Arkin) without undue delay and, where applicable, to the accredited ethics committee in accordance with national regulations. There are no restrictions for concomitant care or interventions. Participants are free to receive any form of additional care as needed.

If a participant and their therapist decide to modify or discontinue the allocated treatment for clinical reasons, this does not lead to withdrawal from the study. Participants will be asked to continue all follow-up assessments and will remain included in study analyses according to the intention-to-treat principle. The sponsor or investigator may discontinue a participant’s study involvement only if continuation of study procedures could affect participant safety. Such decisions do not affect the participants’ access to clinical treatment. All protocol deviations are documented.

### Experimental conditions: blended treatment (Blended-13 and Blended-8)

Patients in Blended-13 and Blended-8 receive an optimised form of blended CBT treatment with MI elements at Jellinek. This blended treatment combines in person or video call therapy sessions with a therapist and therapist-guided digital modules delivered through the secure Karify digital health platform (compliant with NEN 7510/ISO 27001 and General Data Protection Regulation (GDPR) standards). The primary goal of treatment is to support patients in achieving either abstinence or controlled alcohol use (in moderation), usually after a period of abstinence.

For the OptiBlend trial, the digital modules of the blended treatment at Jellinek were optimised and enriched to include modules about nutrition and sleep. This optimised form of blended treatment was developed and refined through six focus groups and 13 one-on-one interviews with current or former AUD patients. Feedback highlighted three main themes: (1) the need for understanding, support and encouragement; (2) clearer guidance with concrete examples and (3) autonomy and personalisation. The modules were revised accordingly by incorporating experts-by-experience stories, additional examples of possible answers and affirming feedback. To increase autonomy and personalisation, several modules were made optional, allowing participants to choose which modules to complete. All adjustments were evaluated and approved by the clinicians at Jellinek.

In both Blended-13 and Blended-8, patients receive the optimised blended treatment. The treatment conditions, however, differ in the intensity of sessions with a therapist. Patients in Blended-13 receive the optimised blended treatment, with 13 weekly therapy sessions alongside therapist-guided digital modules in the Karify platform. Patients in Blended-8 receive the optimised blended treatment, with fewer therapy sessions (eight sessions delivered weekly or biweekly). In this condition, patients complete more of the treatment independently on the digital platform. If clinically indicated, as decided by the therapists, the number of therapy sessions can be increased to a maximum of 13. The optimised blended treatment consists of three phases:

*Pre-treatment phase (approximately 1–4 months, depending on waiting times at different treatment sites):* between intake and the first therapy session, patients have access to the Karify platform’s welcome phase. Here, they can explore a library of information about alcohol use and the treatment programme, and they can start registering their alcohol consumption.

*Treatment phase (3–5 months):* patients receive 8 (Blended-8) or 13 (Blended-13) weekly therapy sessions, complemented by digital modules on the Karify platform. Each session lasts approximately 45 min and starts with a review of recent alcohol use and homework exercises, followed by thematically structured therapeutic content similar to TAU described in the next section. The digital content is supplemented with psychoeducational material, videos, interactive exercises, structured assignments and the aforementioned innovations (eg, experts-by-experience stories and more autonomy). Additionally, two newly developed modules were integrated in this phase:

1. **Nutrition module:** focuses on healthy eating and drinking behaviour following Dutch dietary guidelines^60^ with psychoeducation on the link between nutrition and alcohol use, supported by a food diary in which patients can register their food intake, and optional submodules on setting goals for healthy eating, reasons for and consequences of unhealthy eating, healthier alternatives, energy balance and coping with high-risk situations for unhealthy eating.
2. **Sleep module:** covers psychoeducation on sleep, insomnia and the relationship between sleep and AUD, supported by a sleep diary, and optional submodules on setting goals for sleeping better, healthy sleep behaviours, stimulus control and sleep restriction, reducing worrying, relaxation and dysfunctional sleep-related thoughts.

Patients can access the nutrition and sleep modules at any point throughout the blended treatment and are encouraged to do so by their therapist. If appropriate or necessary during treatment, the contents of these modules will be covered during the therapy sessions as well.

Between sessions, patients work on digital exercises about these themes in Karify. Therapists provide weekly feedback on the completed digital exercises, typically including reinforcement of engagement, content-related comments, follow-up questions or suggestions, and motivational encouragement. Patients are asked to bring a significant other during one of the sessions if possible.

*Aftercare phase (6 months):* patients receive monthly telephone check-ins with their therapist and retain full access to the Karify platform. In addition, an optimised aftercare module will be available, focusing on self-monitoring of alcohol use, motivation, quality of life and social support. This module is self-guided, with no therapist involvement. Patients can select topics relevant to them and record alcohol use daily, weekly or monthly, according to their preference.

### Control condition: treatment as usual

TAU is an individual form of CBT with MI elements and consists of in person or video call sessions with a therapist, assisted by homework assignments using a printed workbook.^61^ TAU is an evidence-based form of AUD treatment that is widely implemented in addiction care in the Netherlands. It is recommended in the Dutch multidisciplinary guideline for the treatment of AUD.^10^ The primary goal of the treatment is to support patients in achieving either abstinence or controlled drinking, usually after a period of abstinence. TAU at Jellinek consists of the same treatment phases as Blended-13 and Blended-8:

*Pre-treatment phase (approximately 1–4 months, depending on waiting times at different treatment sites):* between intake and the first therapy session, patients have access to the Karify platform’s welcome phase. Here, they can explore a library of information about alcohol use and the treatment programme, and they can start registering their alcohol consumption.

*Treatment phase (3–5 months):* patients receive up to 13 weekly individual therapy sessions combined with homework assignments using a printed workbook. Each session lasts approximately 45 min and starts with a review of recent alcohol use and homework exercises, followed by thematically structured therapeutic content. The following themes are addressed: (1) motivation, (2) treatment goals, (3) risk situations, (4) functional analysis, (5) relapse prevention strategies, (6) coping with craving, (7) restructuring of thoughts, (8) communication training focusing on saying no to alcohol/drugs, (9) evaluation of the treatment course and (10) optional exercises on social skills, mood regulation, stress management, problem solving and relapse coping. Patients are asked to bring a significant other during one of the sessions if possible. Between sessions, patients work on homework exercises on these themes in a printed workbook.

*Aftercare phase (6 months):* patients receive monthly telephone check-ins with their therapist.

### Adherence to interventions

To promote adherence to the allocated interventions, several strategies will be implemented. Therapists will be trained in the various interventions and study procedures of the OptiBlend trial before the start. Clear and consistent communication with participating therapists will be maintained throughout the study to ensure understanding of study procedures and the importance of following the protocol. The research team will remain available for questions or reporting of protocol deviations. Intervention adherence will be monitored at scheduled evaluation points and at the end of treatment by reviewing electronic patient records and asking therapists to reflect on intervention delivery and any deviations.

### Sample size

#### Randomised controlled trial

The target sample size is 298 participants, with approximately 99 participants allocated to each of the three treatment arms. The sample size was calculated to detect a small-to-medium between-group effect on the primary outcome, weekly alcohol consumption at 12-month follow-up, comparing the combined blended conditions (Blended-13+Blended-8) with TAU. We assumed an effect size of Cohen’s *d*=0.35, informed by previous literature which reported an effect size of Hedges’ *g*=0.30 for digital CBT in addition to TAU for AUD and *d*=0.41 for blended treatment in other psychiatric disorders.^57 62^ Given the absence of previous RCTs evaluating the present blended treatment format for AUD, *d*=0.35 was considered a realistic effect size.

The calculation was based on a repeated-measures design with assessments at baseline and 3, 6, 12 and 18 months, assuming an intraperson correlation of *ρ*=0.70, a two-sided α of 0.05 and 80% power. The calculation also accounted for clustering by treatment site and an anticipated attrition rate of 20%.

For the non-inferiority comparison of Blended-8 with Blended-13, a non-inferiority margin of 10% of the expected reduction in weekly alcohol consumption was prespecified. This corresponds to 3.5 standard units of alcohol (*SD*=10), based on an expected reduction of approximately 35 standard units per week between baseline and the 12-month follow-up.

#### Ecological momentary assessment

For the EMA study, approximately 100 participants will be recruited from the main trial, assuming equal distribution across the three trial arms. Participants will complete three EMA waves at baseline, 8 weeks and 6 months, each consisting of seven consecutive days, resulting in a maximum of 21 daily measurements per participant.

This sample size was based on the ability to detect medium-sized between-group differences (*d*≥0.60) in nutrition and sleep-related behaviours between the combined blended treatment conditions and TAU. The calculation assumed an intraperson correlation coefficient of *ρ*=0.70 and allowed for missing EMA observations and an anticipated attrition rate of 20%.

#### Recruitment and informed consent

All patients who complete an intake at Jellinek and meet the inclusion criteria are asked by their therapist for permission to be contacted by the research team. If permission is granted, a member of the research team contacts the patient and provides both verbal and written information about the study, including the informed consent form (see online supplemental material 1). Patients are given at least 1 week to consider participation before providing consent. The informed consent appointment takes place either via video call or in person at one of the Jellinek locations. During this appointment, patients receive a detailed explanation of the study procedures and have the opportunity to ask questions.

Informed consent is obtained by trained research staff, all of whom have completed at least essential Good Clinical Practice training. Participants can sign the informed consent form digitally via DocuSign, a secure electronic signature platform, or on paper if preferred. After the participant has signed the form, the research staff member conducting the appointment countersigns it. Both the participant and the research team receive a copy of the fully signed informed consent form. Informed consent is obtained before any study procedures, including baseline assessments.

Participants are asked separately whether they wish to participate in the add-on EMA study, which involves monitoring of nutrition, sleep and alcohol use. Participation in the EMA study is voluntary and does not affect eligibility for the main study. In addition, participants are explicitly asked whether they consent to the use of their data for ancillary or future related studies. This consent is optional and separate from the main study consent. Recruitment started in December 2024 and is ongoing. Completion of recruitment is expected in December 2026.

### Data collection and participant timeline

The baseline assessment (T0) will be conducted before the start of treatment, either in person or via video call, depending on participant preference. During this assessment, self-report questionnaires will be administered digitally through Castor EDC. For participants who opt into the EMA study, the Avicenna smartphone application (previously known as Ethica Data) will be installed, followed by completion of a brief practice questionnaire, after which the first wave begins. Each EMA wave lasts seven consecutive days and is conducted at three time points: during the first week after T0 (wave 1), after 8 weeks of treatment (wave 2) and at the 6-month follow-up (wave 3). All waves consist of the same questions assessing nutritional behaviour (dietary consumption and eating patterns) and sleep (sleep quality and total hours of sleep). Follow-up questionnaire assessments via Castor EDC take place at 3 months (T1), 6 months (T2), 12 months (T3) and 18 months (T4) after T0. The full schedule of enrolment, interventions and assessments is presented in table 1 in accordance with the SPIRIT guidelines.

**Table 1:** Participant timeline in accordance with the SPIRIT guidelines

Study period						
	**Enrolment**	**Allocation**	**Post-allocation**			
**Timepoint**		**T0**	**T1**	**T2**	**T3**	**T4**
		*Week 0*	*Month 3*	*Month 6*	*Month 12*	*Month 18*
**Enrolment**						
Screening	X					
Contact	X					
Informed consent		X				
Allocation		X				
**Interventions**						
Blended-13			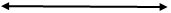			
Blended-8			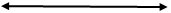			
TAU			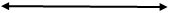			
**Assessments**						
Sociodemographic variables		X				
TLFB		X	X	X	X	X
MATE-Q		X	X	X	X	X
AUDIT		X	X	X	X	X
EQ-5D-5L		X	X	X	X	X
MANSA		X		X	X	X
TiC-P		X		X	X	X
PHQ-9		X		X	X	X
GAD-7		X		X	X	X
PSQI		X		X	X	X
Mini-EAT		X		X	X	X
OCDS		X		X	X	X
AASES-20		X		X	X	X
LPFS-BF 2.0		X				
J-PTSD		X				
GSE		X				
UPPS-P-20		X				
WAI-SR			X			
CSQ-8				X		
SUS				X		

AASES-20, Alcohol Abstinence Self-Efficacy Scale-20; AUDIT, Alcohol Use Disorders Identification Test; CSQ, Client Satisfaction Questionnaire; EQ-5D-5L, EuroQol 5D-5L; GAD-7, Generalized Anxiety Disorder-7; GSE, General Self-Efficacy Scale; J-PTSD, Jellinek Post-Traumatic Stress Disorder Screening Questionnaire; LPFS-BF 2.0, Level of Personality Functioning Scale – Brief Form 2.0; MANSA, Manchester Short Assessment Quality of Life; MATE-Q, Measurement of Addiction for Triage and Evaluation Questionnaire; Mini-EAT, Mini Eating Assessment Tool; OCDS, Obsessive Compulsive Drinking Scale; PHQ-9, Patient Health Questionnaire-9; PSQI, Pittsburgh Sleep Quality Index; SPIRIT, Standard Protocol Items: Recommendations for Intervention Trials; SUS, System Usability Scale; TIC-P, Treatment Inventory of Costs in Patients with Psychiatric Disorders; TLFB, Timeline Follow-back; UPPS-P-20, Urgency Premeditation Perseverance Sensation Seeking Positive Urgency – Short Version; WAI-SR, Work Alliance Inventory – Short Revised Form.

#### Primary outcome measure

##### Timeline Follow-back 7 days

Alcohol consumption will be assessed using the Timeline Follow-back (TLFB) method. The self-report 4-week TLFB (28 or 30 days) is widely used and demonstrates good concurrent validity with diaries of alcohol use and the Alcohol Use Disorder Identification Test (AUDIT), as well as good test-retest reliability.^63–65^

Further evidence indicates that shorter recall periods provide more accurate day-to-day estimates. Although summary indicators derived from the 7-day and 30-day TLFB correspond well, the 7-day TLFB yields more accurate reporting of total consumption, heavy drinking days and abstinent days.^66^ Based on these findings, the 7-day TLFB will be used as the primary outcome measure of alcohol use in the current study. To facilitate comparison and secondary analyses, the 4-week (28-day) TLFB will also be administered for the secondary outcome measure.

### Secondary outcome measures

#### WHO risk drinking levels

The 4-week (28-day) TLFB will be used to calculate changes in World Health Organization (WHO) risk drinking levels from baseline (T0) to post-treatment (6-month follow-up; T2) and to longer-term follow-ups (12-month and 18-month follow-ups; T3 and T4). WHO risk drinking levels classify alcohol-related health risk into low, medium, high or very high categories. Reductions in these risk levels indicate clinically meaningful improvements in functioning and well-being, including fewer alcohol-related consequences and improved quality of life.

#### Measurement of Addiction for Triage and Evaluation Questionnaire

The Measurement of Addiction for Triage and Evaluation Questionnaire (MATE-Q)^67^ will be used to assess treatment response at the 12-month follow-up (T3). Treatment response is defined as successful if, during the 30 days preceding the assessment, participants meet both of the following criteria:

1. No more than eight alcohol drinking days.
2. No heavy drinking days (defined as >3 standard drinks/day for females and >4 standard drinks/day for males).

In addition to alcohol use, the MATE-Q will also be used to monitor the use of other substances, gambling behaviours and alcohol-related craving, as measured by the five-item Obsessive-Compulsive Drinking Scale (OCDS) included within the questionnaire. Higher scores on the OCDS indicate greater craving intensity and loss of control over alcohol use.

The MATE-Q is a self-administered and shortened version of the MATE, designed for individuals with substance use disorder to monitor and evaluate treatment progress and outcomes. The original MATE is a semistructured interview lasting approximately 45–60 min, which has demonstrated good concurrent validity with established measures such as the WHO Disability Assessment Schedule II (WHODAS-II) and acceptable test-retest reliability.^67^ The MATE-Q has shown acceptable concurrent validity when compared with the MATE,^68^ while being less resource-intensive and more feasible for clinical research. However, its reliability has not yet been examined.

#### The Alcohol Use Disorders Identification Test

The Alcohol Use Disorders Identification Test (AUDIT) is a screening instrument for alcohol-related problems over the past 12 months.^69^ The brief version is used, which consists of 8 items scored on a 5-point scale and two items scored on a 3-point scale. A score of 8 or higher indicates further diagnostic research. The brief version of the AUDIT shows good to excellent test-retest reliability (intraclass correlation coefficient (ICC) =0.84) and good construct validity according to a systematic review.^70^

### Measures for economic evaluation

#### EuroQol 5D-5L

The EuroQol 5D-5L (EQ-5D-5L)^71^ is a standardised instrument for measuring health-related quality of life across five dimensions (eg, mobility, self-care, usual activities, pain/discomfort, anxiety/depression) plus a Visual Analogue Scale (VAS) for self-rated health. Each dimension is measured on a 5-point scale, ranging from *1=No problems* to *5=Unable* to or *Severe problems*. The VAS ranges from 0 (worst imaginable health) to 100 (best imaginable health). The EQ-5D-5L demonstrates excellent psychometric properties, including high test-retest reliability and acceptable construct and across-group validity.^72^

#### Treatment Inventory of Costs in Patients with Psychiatric Disorders

The Treatment Inventory of Costs in Patients with Psychiatric Disorders (TiC-P) measures healthcare consumption and productivity loss in patients with psychiatric disorders.^73 74^ It consists of 57 items, allowing for the economic evaluation of mental illness and healthcare consumption.^74^ The questions refer to the past 3 months.

For the present study, two of the follow-up assessments (T3 and T4) occur at 6-month intervals. To obtain a comprehensive picture of participants’ use of addiction care, an additional question will be added to the TiC-P asking whether participants have received addiction treatment during the past 6 months. This information is important for evaluating possible relapse and the effectiveness of the aftercare module in the study. The TiC-P has demonstrated satisfactory construct validity and test-retest reliability for estimating work absence and the number of contacts with healthcare providers. However, reliability is lower for reporting contact with medical specialists or alternative practitioners.^74^

#### Other measures

Other measures described below will be used to describe the sample, explore potential predictors or moderators and support secondary or exploratory analyses.

#### Sociodemographic characteristics

At baseline (T0), participants will complete a questionnaire assessing demographic and background characteristics, including sex, age, country of birth of both parents, education level, employment status, marital status, number of children, living situation, housing conditions, history of addiction and psychiatric treatment, and family history of addiction.

#### Manchester Short Assessment Quality of Life

The Manchester Short Assessment Quality of Life (MANSA)^75^ is a brief self-report questionnaire designed to assess quality of life across multiple life domains (eg, life, work and personal safety). It consists of 12 items rated on 7-point Likert scales, ranging from *1=Not at all satisfied* to *7=Extremely satisfied,* and 4 items answered with a yes/no response. The MANSA has demonstrated good internal consistency (α=0.84) and good convergent and divergent validity.^76^

#### Patient Health Questionnaire

The Patient Health Questionnaire (PHQ-9)^77^ is a brief, self-administered screening tool for assessing depressive symptom severity over the past 2 weeks. It consists of nine items rated on a 4-point scale ranging from *0=Not at all* to *3=Nearly every day*. A higher score indicates more depressive symptoms. The PHQ-9 generally demonstrates good internal consistency (α=0.86), though reporting of test-retest reliability and inter-rater reliability is limited.^78^ In terms of validity, most studies report sensitivity and specificity (approximately 76% each), with moderate levels of convergent validity. A recent study examined the validity of the PHQ-9 in individuals with substance use disorders, supporting its validity and factor structure in this population.^79^

#### Generalized Anxiety Disorder-7

The Generalized Anxiety Disorder-7 (GAD-7)^80^ is a self-report questionnaire used to screen for and assess the severity of generalised anxiety disorder (GAD) symptoms over the past 2 weeks. It consists of seven items, rated on a 4-point scale ranging from *0=Not at all* to *3=Nearly every day*. A higher score indicates more GAD symptoms. The GAD-7 has demonstrated excellent internal consistency (α=0.92) and good test-retest reliability (ICC=0.83). It also shows strong criterion validity, with a sensitivity of 89% and specificity of 82% for detecting GAD, as well as good construct validity.^80^ The validity and factor structure of the GAD-7 were further supported among individuals with substance use disorders,^79^ demonstrating that the measure is appropriate for use in this population.

#### Pittsburgh Sleep Quality Index

The Pittsburgh Sleep Quality Index (PSQI)^81^ is a widely used instrument for assessing sleep quality in both clinical and research settings. It consists of 24 items, of which 19 are self-reported and usually used to calculate the total score. These items cover seven aspects of sleep, including sleep quality, sleep duration, sleep latency, sleep efficiency, sleep disturbances, use of sleep medication and daytime dysfunction. The remaining five items can be completed by a roommate or bed partner when applicable, but are not included in the total score. Each item is rated on a 0–3 scale, with higher scores indicating more frequent sleep problems. The seven component scores are summed up to produce a global score ranging from 0 to 21, where higher scores reflect poorer sleep quality. A systematic review and meta-analysis found the PSQI to have good reliability (α=0.70–0.84) and construct validity across diverse populations.^82^

#### Mini Eating Assessment Tool

The Mini Eating Assessment Tool (Mini-EAT)^83^ is a brief, nine-item dietary screener designed for use in clinical and research settings to quickly assess diet quality. It measures the consumption of fruits and vegetables, whole grains, refined grains, fish or seafood, legumes, nuts and seeds, low-fat dairy, high-fat dairy and sweets. The Mini-EAT was developed as a shortened version of the 19-item Eating Assessment Tool to provide a practical alternative to comprehensive dietary assessments such as the Healthy Eating Index (HEI-2015), which is based on a 156-item food frequency questionnaire. Validation analysis demonstrated good concurrent validity with HEI-scores (*r*=0.71).^83^ However, the reliability of the Mini-EAT has not yet been examined.

#### Alcohol Abstinence Self-Efficacy Scale-20

The Alcohol Abstinence Self-Efficacy Scale-20 (AASES-20)^84^ is a self-report questionnaire that measures an individual’s confidence about their ability to avoid drinking. It consists of 20 items rated on a 5-point Likert scale, ranging from *1=Not at all confident* to *5=Extremely confident*. The internal consistency of the AASES-20 is excellent (α=0.92).^84^ The validity of the AASES has not yet been examined.

#### Level of Personality Functioning Scale – Brief Form 2.0

The Level of Personality Functioning Scale – Brief Form 2.0 (LPFS-BF 2.0)^85^ is a brief self-report questionnaire for assessing personality functioning. It consists of 12 items, rated on a 4-point Likert scale ranging from *1=Very false or often false* to *4=Very true or often true.* As a result, participants can achieve a total score between 12 and 48, with higher scores indicating greater severity of impairment in personality functioning. The LPFS-BF 2.0 has demonstrated acceptable internal consistency (α=0.82) and construct validity.^85^

#### Jellinek PTSD Screening Questionnaire

The Jellinek Post-Traumatic Stress Disorder Screening Questionnaire (J-PTSD)^86^ is a brief screening instrument for PTSD. It is a modified version of the Primary Care PTSD Screen.^87^ In the first question of the screener, participants are asked to mark all traumatic events (eg, serious accident, rape, sexual abuse or other) they have experienced. If no traumatic event is reported, the questionnaire ends. Participants who report trauma complete four yes/no items assessing re-experiencing, avoidance, hyperarousal and numbing symptoms. A cut-off score of 2 is used to indicate possible PTSD. The J-PTSD has demonstrated high sensitivity (0.87), specificity (0.75) and overall efficiency (0.77) for detecting PTSD.^86^ The reliability of the J-PTSD has not yet been examined.

#### General Self-Efficacy Scale

The General Self-Efficacy Scale (GSE)^88^ is used to measure a person’s general sense of self-efficacy in handling challenging situations. The scale consists of 10 items, rated on a 4-point Likert scale ranging from *1=Not at all true* to *4=Exactly* *true*. Higher scores reflect greater general self-efficacy. The GSE scale has demonstrated good to excellent internal consistency (α=0.86–0.94) and good construct validity across multicultural samples.^89^

#### Urgency Premeditation Perseverance Sensation Seeking Positive Urgency - Short Version

The Urgency, Premeditation, Perseverance, Sensation Seeking, Positive Urgency - Short Version (UPPS-P-20)^90^ is a self-report questionnaire assessing impulsivity using the five-factor model. It consists of 20 items, rated on a 4-point Likert scale ranging from *1=Totally agree* to *4=Totally disagree*. The scale evaluates five dimensions of impulsivity: Urgency, Positive Urgency, Lack of Premeditation, Lack of Perseverance and Sensation Seeking. A higher score indicates greater impulsivity on that dimension. The UPPS-P-20 has demonstrated good internal consistency (α=0.70–0.84) and external validity.^90^

#### Working Alliance Questionnaire – Short Revised

The Working Alliance Inventory (WAI)^91^ is the most widely used instrument for assessing therapeutic alliance. The short, revised version, the Working Alliance Questionnaire – Short Revised (WAI-SR),^92^ consists of 12 items, each rated on a 5-point Likert scale, ranging from *1=Seldom or never* to *5=Always*. The WAI-SR was developed to offer a more practical and efficient alternative to the WAI. Research shows that the WAI-SR has psychometric properties comparable to the full WAI. It demonstrates good to excellent internal consistency (α=0.88–0.92) and evidence for construct validity.^92^

#### Client Satisfaction Questionnaire

The Client Satisfaction Questionnaire (CSQ-8)^93^ is an eight-item questionnaire designed to assess overall patient satisfaction with mental healthcare services. Items are rated on a 4-point ordinal scale, with higher scores indicating greater satisfaction. The Dutch translation of the CSQ-8 has been validated and demonstrates high internal consistency and good concurrent validity in mental healthcare settings, including among individuals with substance use disorder (SUD).^94 95^

#### System Usability Scale

The System Usability Scale (SUS)^96^ is a widely used questionnaire that assesses perceived usability of tools or systems. It consists of 10 items, rated on a 5-point Likert scale from *1= Strongly disagree* to *5=Strongly agree*. Odd-numbered items are positively worded, whereas even-numbered items are negatively worded. After scoring and standard SUS transformation, total scores range from 0 to 100, with higher scores reflecting better perceived usability. The SUS demonstrated adequate internal consistency (α=0.74), good test-retest reliability and acceptable construct validity.^97^ In the present study, the SUS is used to assess the usability of the Karify platform.

#### Plans to promote participant retention and complete follow-up

To support participant engagement and maximise follow-up completion, several retention strategies are implemented. At baseline (T0), assessments are conducted in person or via video call, depending on participant preference. During this session, a member of the research team is available to address questions and provide support.

For all follow-up assessments, participants are contacted by telephone in advance to notify them that the questionnaires will be sent and to ensure that the questionnaire is successfully received by email. Participants are encouraged to complete the questionnaires within the following days, and assistance is offered if needed.

Finally, participants receive a reimbursement of €20 for each completed measurement (T0–T4) in the form of a voucher. Those who take part in the EMA study receive an additional financial compensation of €2 per completed EMA measurement, with a maximum of €14 per wave. In total, participants can earn up to €100 for completing all RCT measurements and up to €142 if they also participate in the EMA study.

### Data management

All study data will be collected and stored in Castor EDC, which complies with European Union regulations for clinical research data management, including GDPR and the applicable information security standards (ISO/IEC 27001/2 and NEN 7510). A data processing agreement has been established between Castor EDC and the study sponsor. Data are stored on European servers, and each participant is assigned a unique study identification number. No directly identifiable personal data are entered into Castor EDC, and a full audit trail of all data operations is maintained.

EMA data will be collected using the Avicenna Research smartphone application. Avicenna complies with GDPR requirements, and a data processing agreement was signed between Avicenna Research and the study sponsor. Data collected through Avicenna are stored on secure Canadian servers and are pseudonymised: participants use their study ID and a study-specific email address, and an additional Avicenna ID is generated.

To enable linkage between datasets, a key file containing study IDs, Castor IDs and Avicenna IDs is stored separately on a secure, access-restricted drive within Arkin. The file is password-protected and accessible only to authorised research staff. All pseudonymised study data will be stored for up to 10 years after trial completion.

### Statistical methods

All analyses follow the Consolidated Standards of Reporting Trials reporting guidelines for RCTs, the Consolidated Health Economic Evaluation Reporting Standards and Dutch guidelines for economic evaluation.^98^ Analyses will be performed on the full randomised sample according to the intention-to-treat principle. Additionally, per-protocol sensitivity analyses will include only participants who completed a substantial proportion of their allocated intervention. Analyses will be conducted in R or SPSS. Missing data will be handled using multiple imputation (R package *mice* or equivalent).

Intervention effects will be analysed using (robust) linear mixed models or generalised linear mixed models ((G)LMMs), with link functions chosen according to the type of treatment outcome variable (continuous/count data for alcohol use, ordinal/categorical data for WHO risk drinking levels, and dichotomous data for treatment response). The variable *Treatment group* will be included as a fixed effect in all models and will be coded according to the specific analysis. Participants will be included as random effects to account for repeated or nested measures. For longitudinal analyses, the categorical variable *Time* (Baseline, 3 months, 6 months, 12 months (primary endpoint) and 18 months) will be included, and the interaction *Treatment group × Time* will be examined to assess whether intervention effects differ across follow-up points. The stratification variable, *Trial site*, will be included as a covariate in all models.

### Primary outcome

The primary objective is to evaluate the effectiveness of the two blended treatment conditions combined (Blended-8+Blended-13) compared with TAU in reducing weekly alcohol consumption at the 12-month follow-up. For the primary analysis, a robust LMM (eg, using the R package *robustlmm* or equivalent) will be used to compare the treatment groups (Blended-8+Blended-13 vs TAU) at this single time point (12 months).

### Secondary outcome

#### Non-inferiority analysis

For the second hypothesis, the non-inferiority of Blended-8 to Blended-13 in reducing weekly alcohol consumption at the 6-month and 12-month follow-ups is evaluated. To do this, a robust LMM will be used, restricted to comparing the two blended treatment conditions. Non-inferiority will be concluded if the 90% CI of the estimated treatment effect stays within the predefined 10% non-inferiority margin.

#### Bayesian analyses

In addition to the primary and secondary frequentist analyses, exploratory Bayesian analyses will be conducted. Bayesian linear mixed models will be specified in line with the primary models, using weakly informative priors. Posterior distributions will be used to estimate the probability of treatment effects, including the probability that the combined blended conditions are more effective than TAU, and that Blended-8 is non-inferior to Blended-13. Where appropriate, Bayes factors will be reported to quantify the relative evidence for competing hypotheses. These analyses are considered complementary and will be used to support the interpretation of the primary findings.

#### Economic evaluation

For the third hypothesis, the cost-effectiveness of all comparisons between treatment conditions (Blended-8 vs Blended-13, Blended-8 vs TAU, and Blended-13 vs TAU) is evaluated. Costs include the following: (1) intervention delivery, (2) general healthcare uptake including medication, (3) patient out-of-pocket expenses and (4) productivity losses (due to absenteeism or presenteeism).

Cost data will be collected using the TiC-P questionnaire, and productivity losses will be calculated based on gender-specific and age-specific friction costs. The Dutch guideline for economic evaluation^98^ will be followed, and standard cost prices reported therein will be applied. Health outcomes will include both the primary outcome (weekly alcohol consumption) and quality-adjusted life years (QALYs) derived from the EQ-5D-5L questionnaire using Dutch tariffs. The area under the curve method will be applied to weight utility scores across follow-up measurement waves, enabling the computation of cumulative QALYs.

The incremental cost-effectiveness ratio (ICER) will be calculated for all comparisons between treatment conditions. CIs for the ICER will be estimated using a non-parametric bootstrap and a net benefit regression approach. ICERs will be displayed on cost-effectiveness planes, and cost-effectiveness acceptability curves will be plotted. Sensitivity analyses targeting uncertainty in the main cost drivers will be conducted to assess the robustness of the key findings.

#### Sleep and nutrition outcomes

For the fourth hypothesis, the additional effects of the nutrition and sleep modules in the blended interventions will be evaluated at 6 months using the EMA, PSQI and Mini-EAT. Analyses will be conducted using a robust LMM. Daily EMA observations, collected over seven consecutive days at each time point (baseline, 8 weeks, 6 months), will be nested within participants. The variable treatment group combines the blended conditions (Blended-8+Blended-13) compared with TAU. The primary estimates of interest are the between-group differences in changes in sleep quality and dietary behaviour over time, as measured by both the daily EMA data and the PSQI and Mini-EAT at 6 months. The 8-week EMA data will be used as an intermediate measure to explore early changes.

#### Other outcomes

For the WHO risk drinking levels, an ordinal mixed-effects regression model will be fitted (eg, using the R package *MASS* or equivalent). For the treatment response data, a generalised mixed model with a binomial link function will be used to indicate whether treatment has been successful. This will be done using the R package *lme4* or equivalent.

### Ethics and dissemination

Ethical approval for the conduct of the trial was issued by the METC Amsterdam UMC (NL84117.018.23) on 17 October 2024. Amendments will be reported to the METC Amsterdam UMC. All participants provide written informed consent before participating in the trial.

The trial sponsor monitors data through internal and external audits, in compliance with GDPR requirements. In addition, audit trails are generated to foster data integrity. No independent Data Monitoring Committee has been appointed for this study, as the risks associated with participation are considered negligible. No interim analyses are planned, as no safety concerns are anticipated that would require early termination of the trial.

### Patient and public involvement

Patients were actively involved throughout the OptiBlend study to ensure that the trial design, procedures and interventions aligned with patient needs and preferences. During the design phase, focus groups and interviews with former and current Jellinek patients were conducted to create the new and optimised modules of the blended treatment programme. Also, the Arkin patient council reviewed the grant application, study design and recruitment procedures to ensure the trial is implemented in a patient-centred and ethically responsible manner.

During and after completion of the study, key findings will be summarised in lay language and disseminated through Jellinek’s website and newsletters. Participants who express interest will receive a lay summary of the outcomes, including practical implications. Finally, the study results will be implemented at Jellinek’s outpatient facilities, if deemed appropriate after the RCT results are available, directly benefiting future patients.

## Discussion

The OptiBlend trial is among the first studies designed to evaluate the effectiveness and cost-effectiveness of an optimised blended treatment for AUD. In this three-arm, single-blinded RCT conducted at four outpatient facilities of Jellinek, we compare TAU (13 therapy sessions of CBT/MI treatment) to two forms of optimised blended treatment: one with the same number of therapy sessions as TAU (Blended-13) and one with fewer therapy sessions (Blended-8), both enriched with digital modules targeting nutrition and sleep. The trial will assess whether clinical effectiveness can be maintained with fewer therapy sessions in blended treatment, potentially improving cost-effectiveness. We will also examine the added value of targeting lifestyle factors (ie, nutrition and sleep) that are frequently disrupted in AUD and linked to relapse risk.^4 6–8^ To our knowledge, this is the first RCT in addiction care to examine whether a blended treatment can achieve similar clinical effectiveness with reduced therapist contact.

Blended treatment combines the strengths of traditional treatment and digital interventions, potentially addressing limitations of both approaches, including high relapse, low adherence and a substantial treatment gap.^15 22 23 30^ Therapy sessions may enhance motivation and engagement,^35 36^ while digital modules offer flexibility and autonomy, allowing patients to complete parts of treatment independently and potentially reducing the number of therapy sessions required. This approach may reduce waiting times, reliance on trained healthcare professionals and overall healthcare costs.^39^

The OptiBlend trial is expected to provide insights into multiple domains. We hypothesise that both blended interventions will lead to greater reductions in alcohol consumption compared with TAU, with Blended-8 being non-inferior to Blended-13. From a societal perspective, Blended-8 may be more cost-effective than both Blended-13 and TAU. Additionally, the study will explore the impact of nutrition and sleep modules, offering novel insights into how to optimise blended interventions and address relapse-related lifestyle factors.

The OptiBlend trial has several strengths. Conducting the study in a real-world clinical setting enhances ecological validity, further supported by EMA. Active involvement of patients and therapists in developing the intervention ensures a patient-centred design and clinical relevance. Moreover, the inclusion of multiple outcome measures, such as clinical outcomes, cost-effectiveness and lifestyle factors, enables a comprehensive evaluation of the blended approach.

Potential limitations should be considered. The study focuses exclusively on AUD and is therefore not generalisable to individuals with other primary SUDs. While outcome assessors are blinded, therapists and participants are not, which could introduce bias. Also, reliance on self-report measures may be subject to reporting biases. Finally, internal validity may be affected by variations in adherence or deviations from the protocol that cannot be fully controlled.

If effective, blended treatment could offer a more accessible, flexible and efficient option for AUD treatment. Reducing the number of therapy sessions may help close the treatment gap and lower healthcare costs. Additionally, results may be informative for the broader implementation of blended interventions in addiction care. Further research could explore the applicability of the blended approach to other SUDs and psychiatric disorders.

In conclusion, the OptiBlend trial addresses key research gaps by evaluating the (cost-)effectiveness of an optimised blended treatment for AUD, with modules targeting nutrition and sleep, and with a reduced amount of therapy sessions. Findings will provide important insights into optimising blended interventions, enhancing treatment accessibility and improving long-term outcomes for individuals with AUD.

### Trial status

The OptiBlend trial is currently recruiting participants. Recruitment started on 9 December 2024 and is ongoing at the time of manuscript submission. As of August 2026, 143 participants have been enrolled. Recruitment is expected to continue until 31 December 2026, and data collection is anticipated to be completed by June 2028. The current protocol version is 06.

## Supplementary material

10.1136/bmjopen-2026-127286 online supplemental file 1

## Data Availability

No data are available.
