# Supplementary material for: Optimised blended treatment for alcohol use disorder (OptiBlend) in Dutch outpatient addiction care: study protocol for a three-arm randomised controlled trial
Source: BMJ Open. 2026 Nov 6;16(11):e127286. doi: 10.1136/bmjopen-2026-127286 (PMC13756488; doi:10.1136/bmjopen-2026-127286)
Supplement: Supplementary file 2 [file bmjopen-16-11-s001.docx]

**Supplemental Material 1**

**Participant Consent Form – English Translation**

**METC reference number**: NL84117.018.23
**Original Dutch document**: Version 6, dated 11 February 2025

*This document is an English translation of the Dutch participant consent form approved for use in the OptiBlend trial. Participants provided informed consent using the Dutch-language version.*

**Participant consent form**

For the OptiBlend study: *Effectiveness of an optimised blended treatment for alcohol use disorder*

- I have read the participant information sheet. I have also had the opportunity to ask questions, and my questions have been answered satisfactorily. I have had sufficient time to decide whether I wish to participate.
- I understand that participation is voluntary. I also understand that I may decide at any time not to participate in the study or to withdraw from the study. I do not have to provide a reason for withdrawing.
- I give the researchers permission to inform my therapist that I am participating in this study.
- I give the researchers permission to inform my therapist if my questionnaire responses indicate concerns about my safety. If I am no longer receiving treatment at that time, I give permission for my general practitioner to be informed.
- I give the researchers permission to collect and use my data. The researchers will use these data only to answer the research questions of this study.
- I understand that, for the purposes of monitoring the study, certain individuals may have access to all my data. These individuals are listed in the participant information sheet. I give these individuals permission to access my data for this purpose.
- I give permission for data from my electronic patient record at Arkin, including diagnoses, to be retrieved from my Jellinek patient record and used for this study.
- I give permission for my data stored in Karify to be retrieved and used for this study.

**Please indicate yes or no for each of the statements below.**

**I give permission to participate in the smartphone app study and for the data collected through the app to be used for this study.**

☐ Yes
☐ No

**I give permission for my contact person to be contacted in order to reach me if I cannot be contacted directly.**

☐ Yes
☐ No

**I give permission for my data to be used for other scientific research in the field of addiction care and the further development of blended treatment.**

☐ Yes
☐ No

**I give permission to be contacted after this study to ask whether I would be interested in participating in a follow-up study.**

☐ Yes
☐ No

- I agree to participate in this study.

**Participant name:** ..............................................................

**Signature:** ..............................................................
**Date:** ____ / ____ / ______

I declare that I have fully informed this participant about the study described above.

If information becomes available during the study that may affect the participant's consent, I will inform the participant in a timely manner.

**Name of researcher (or representative):** ..............................................................

**Signature:** ..............................................................
**Date:** ____ / ____ / ______
